# HIV-1 infections with multiple founders associate with the development of neutralization breadth

**DOI:** 10.1371/journal.ppat.1010369

**Published:** 2022-03-18

**Authors:** Eric Lewitus, Samantha M. Townsley, Yifan Li, Gina C. Donofrio, Bethany L. Dearlove, Hongjun Bai, Eric Sanders-Buell, Anne Marie O’Sullivan, Meera Bose, Hannah Kibuuka, Lucas Maganga, Sorachai Nitayaphan, Fredrick K. Sawe, Leigh Anne Eller, Nelson L. Michael, Victoria R. Polonis, Julie A. Ake, Sandhya Vasan, Merlin L. Robb, Sodsai Tovanabutra, Shelly J. Krebs, Morgane Rolland

**Affiliations:** 1 U.S. Military HIV Research Program, Walter Reed Army Institute of Research, Silver Spring, Maryland, United States of America; 2 Henry M. Jackson Foundation for the Advancement of Military Medicine, Inc., Bethesda, Maryland, United States of America; 3 Makerere University Walter Reed Project, Kampala, Uganda; 4 National Institute for Medical Research-Mbeya Medical Research Center, Mbeya, Tanzania; 5 Armed Forces Research Institute of Medical Sciences, Bangkok, Thailand; 6 Kenya Medical Research Institute/U.S. Army Medical Research Directorate-Africa/Kenya-Henry Jackson Foundation MRI, Kericho, Kenya; 7 Center for Infectious Disease Research, Walter Reed Army Institute of Research, Silver Spring, Maryland, United States of America; University of North Carolina at Chapel Hill, UNITED STATES

## Abstract

Eliciting broadly neutralizing antibodies (bnAbs) is a cornerstone of HIV-1 vaccine strategies. Comparing HIV-1 envelope (env) sequences from the first weeks of infection to the breadth of antibody responses observed several years after infection can help define viral features critical to vaccine design. We investigated the relationship between HIV-1 env genetics and the development of neutralization breadth in 70 individuals enrolled in a prospective acute HIV-1 cohort. Half of the individuals who developed bnAbs were infected with multiple HIV-1 founder variants, whereas all individuals with limited neutralization breadth had been infected with single HIV-1 founders. Accordingly, at HIV-1 diagnosis, *env* diversity was significantly higher in participants who later developed bnAbs compared to those with limited breadth (p = 0.012). This association between founder multiplicity and the subsequent development of neutralization breadth was also observed in 56 placebo recipients in the RV144 vaccine efficacy trial. In addition, we found no evidence that neutralization breath was heritable when analyzing env sequences from the 126 participants. These results demonstrate that the presence of slightly different HIV-1 variants in acute infection could promote the induction of bnAbs, suggesting a novel vaccine strategy, whereby an initial immunization with a cocktail of minimally distant antigens would be able to initiate bnAb development towards breadth.

## Author summary

Vaccines against viral pathogens protect through the induction of broadly neutralizing antibodies (bnAbs). No HIV-1 vaccine has successfully elicited bnAbs, and a successful HIV-1 vaccine will need to accelerate the process of development of a broadly neutralizing response that typically takes a couple of years to develop in natural infection. We studied diversity in the HIV-1 envelope gene from initial infection to several years out in 126 individuals from two cohorts. We showed that the development of bnAbs at 2–3 years was not due to transmissible viral genetics, but rather associated with diversity during the first month of infection. We propose that designing a vaccine that mimics an infection with multiple, minimally distant founder variants may successfully elicit the development of bnAbs and provide effective prophylaxis against HIV-1.

## Introduction

Following HIV-1 infection, the HIV-1 Envelope (Env), which mediates virus entry in T cells through binding to the CD4 coreceptor, is the key target of the humoral response mounted by the host. The initial antibody response against Env is observed about two weeks after the detection of viremia [1]. In the following months, neutralizing antibodies develop against the Env of contemporaneous autologous viruses and progressively broaden to recognize heterologous viruses [2–4]. A few years after infection, approximately half of the individuals had antibodies that recognized half of the viruses in diverse panels, with an increasingly smaller fraction of individuals neutralizing higher proportions of viruses [5–9]. In the past decade, hundreds of broadly neutralizing antibodies (bnAbs) have been isolated from people living with HIV-1 (PLWH) [10]. These bnAbs cover multiple Env targets [11] and are directed to both the Env protein and the glycans linked to it. Some bnAbs are extremely potent, such as CAP256-VRC26.25, which targets the variable loops V1 and V2 and showed a 50% inhibitory concentration (IC50) of 0.001 μg/ml [12]. Multiple studies have shown that broadly neutralizing monoclonal antibodies provide protection against infection in non-human primate challenge models [13–18], indicating that vaccination strategies that elicit similar bnAbs could provide prophylactic protection from HIV-1 infection. While the results of these initial challenge studies were reported twenty years ago, candidate HIV-1 immunogens have not yet succeeded in eliciting bnAbs following immunization in human clinical trials. The most potent and broadest neutralizing antibodies often have unique features, such as high levels of somatic hypermutation or heavy chains with a long third complementarity-determining region (CDR H3), that appear complex to reproduce via current immunization strategies.

The challenge with inducing bnAbs stems in part from our incomplete understanding of their ontogeny. The prevalence of bnAbs among chronically infected individuals suggests that the time-dependent maturation of neutralizing antibodies is associated with continuous antigen exposure and specifically with antigens that diversify over time. In a typical HIV-1 infection, the initial viral population is homogeneous [19] and envelope sequences diversify over time by about 1% per year [20], in part driven by a cycle of escape from contemporaneous immune responses [3,21–23]. Several studies showed that neutralization breadth associates with high viral loads [5,23–25], although high viremia is not an absolute precondition for the development of broadly neutralizing responses [26]: for example, the bnAb VRC01 was isolated from a long term non-progressor who maintained viremia around 10,000 copies/ml [27]. HIV-1 diversity has also been positively associated with neutralization breadth, including in the largest cross-sectional study of variables associated with cross-neutralization (n = 4,484) [24,25]. Moreover, the impact of increased viral diversity on the development of neutralization breadth was highlighted in studies of PLWH who then became super-infected [28–34]. Understanding viral diversity, from the beginning of infection through the development of broadly neutralizing antibodies, can better inform future HIV-1 vaccine strategies to elicit similar responses.

We previously evaluated the breadth and potency of neutralizing antibodies in 73 treatment naïve individuals enrolled in the prospective RV217 cohort [35], and found that individuals who developed bnAbs could be distinguished from those who did not develop bnAbs based on B cell engagement with founder Env in the first months of infection [26]. We previously showed that there was no evidence that participants with more complete Env glycan shields (i.e., with smaller glycan holes) were more likely to develop bnAbs [36]. Here we characterized the relationship between Env genetic features from acute infection through the development of broad neutralizing antibody responses. We analyzed 1,252 *env* sequences sampled in these RV217 individuals within 42 days of HIV-1 diagnosis [37]. Individuals who later developed bnAbs showed higher *env* diversity in acute infection and were more likely to have been infected with multiple HIV-1 founders than individuals who did not develop bnAbs. These data help identify molecular features that could promote bnAbs induction, suggesting that a vaccine strategy relying on minimally distant antigens could be advantageous to the development of a globally effective HIV-1 vaccine.

## Results

### Study characteristics

Participants at increased risk for HIV-1 acquisition were enrolled in the RV217 prospective cohort and twice-weekly tested for HIV-1 infection. Upon HIV-1 diagnosis, participants were followed for up to four years [35]. HIV-1 genomes were sequenced from plasma samples via endpoint dilution, with a median of 10 genomes obtained per time point [37,38]. We analyzed 3,482 *env* sequences sampled from 70 participants prior to antiretroviral treatment, with time points collected within 42 days of diagnosis (0–42 days, median = 7.5 days, IQR = 26 days, n = 1262 sequences from 70 participants), at six months (43–261 days, median = 170.5 days, IQR = 16.5 days, n = 447 sequences from 39 participants), one year (262–532 days, median = 421 days, IQR = 84 days, n = 450 sequences from 12 participants), two years (533–843 days, median = 691 days, IQR = 94 days, n = 520 sequences from 12 participants), and three years (843–2115 days, median = 1097 days, IQR = 233.5 days, n = 813 sequences from 21 participants) post-diagnosis (S1 Table, S1 Fig). These participants were from Kenya (n = 18), Tanzania (n = 16), Uganda (n = 10) and Thailand (n = 26). Neutralization breadth was measured against a diverse panel of 34 viruses using samples collected up to three or four years after infection and prior to antiretroviral therapy [26]. Among 70 RV217 participants with infections sequenced prior to 42 days, 16 could neutralize >70% of the virus panel (referred to as broad neutralizers) while 12 individuals only neutralized <35% of the viral panel (referred to as non-broad neutralizers) despite at least 2.5 years of follow up (the panel of 34 viruses was adapted from a prior study [39]). The last time point was at 1,302 days for broad neutralizers and at 1,143 days for non-broad neutralizers. Among the 16 RV217 participants who developed bnAbs, three individuals were found to be super-infected when neutralization breadth developed (participants 40123, 40134, 40512). In addition to RV217 samples, we investigated the same question in the RV144 cohort which included 110 participants with CRF01_AE infections sequenced at HIV-1 diagnosis [40,41]. Neutralization breadth was determined (using the same 34 virus panel) at one to three years post-diagnosis from 90 of these participants, corresponding to 34 RV144 vaccine and 56 placebo recipients. Broad neutralization, as defined by neutralizing >70% of the viral panel, was only observed among placebo recipients (n = 8) [42].

### No evidence of heritability of neutralization breadth among 126 participants

**Fig 1:**
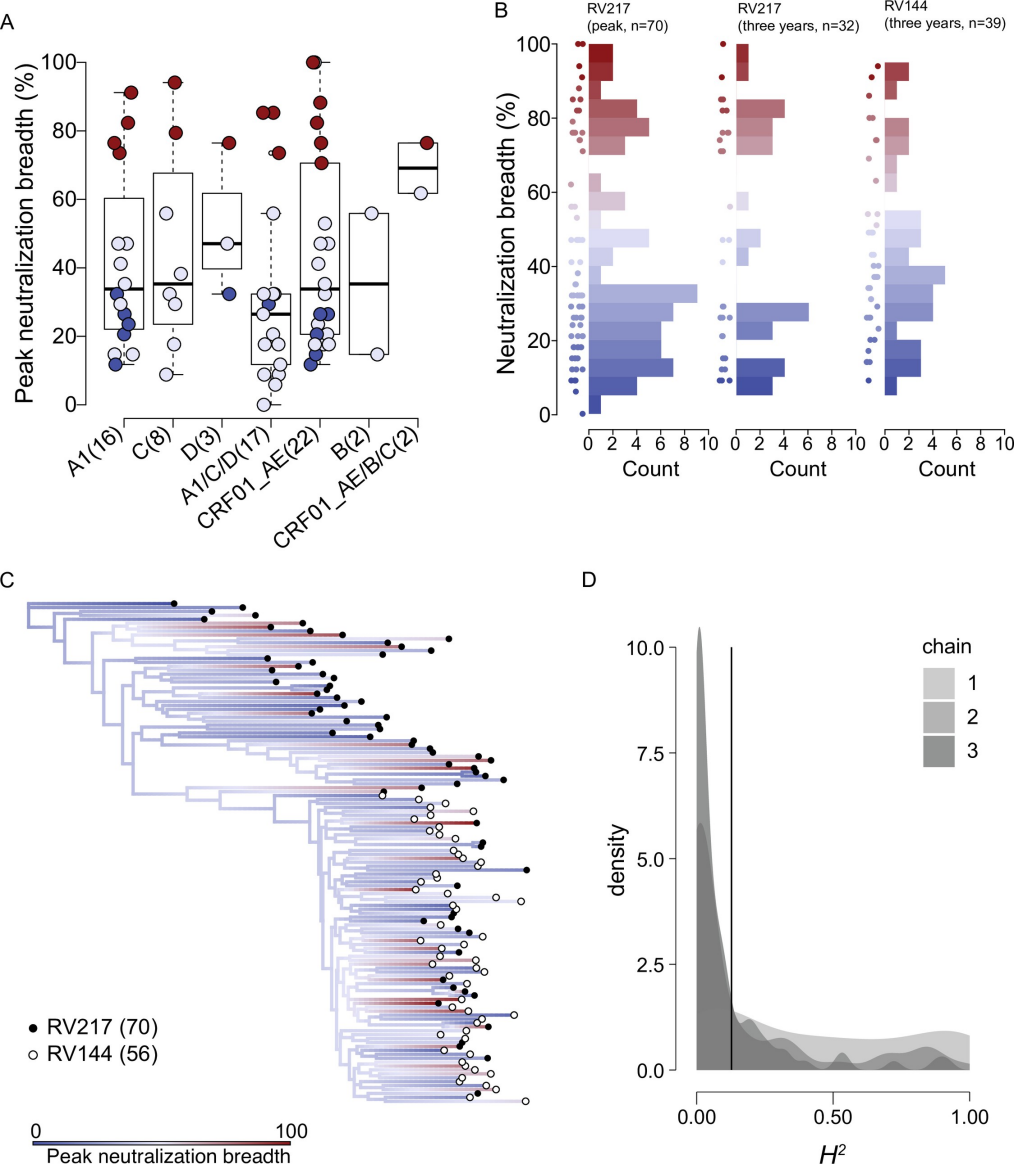
No evidence of heritability of neutralization breadth. Distribution and heritability of peak neutralization breadth across individuals. Non-broad neutralizers (indicated in blue) are defined as individuals who neutralized <35% of a 34-virus panel three to four years post-diagnosis and broad neutralizers (indicated in red) as individuals who neutralized >70% of the panel. Individuals sampled less than two years post-infection (intermediate neutralizers) are also shown (indicated in lavender). (A) Boxplot of peak neutralization breadth across HIV-1 subtypes and recombinants in the RV217 cohort. The number of individuals in each group is shown in parenthesis. (B) Histogram of neutralization breadth measured at peak neutralization breadth (RV217, n = 70) and three years after infection (RV217, n = 32; RV144, n = 39); dots for each participant are shown alongside each histogram. (C) Ancestral reconstruction of neutralization breadth across a phylogeny reconstructed from consensus sequences from the earliest post-diagnosis samples in 70 RV217 individuals (filled circles) and 56 RV144 placebo participants (open circles). The color spectrum shows the reconstructed ancestral estimates for neutralization breadth. (D) Density plot of the H^2^ (heritability) estimates from three conditioned runs (chains) of a phylogenetic Ornstein-Uhlenbeck mixed model performed on the phylogeny in (C). The solid line shows the mean of the posterior estimates of H^2^.

The RV217 cohort comprised individuals living with diverse HIV-1 subtypes. Considering their envelope sequences, these were principally subtype A1 (n = 16) in East Africa along with subtypes C (n = 8) and D (n = 3) and multiple unique recombinants forms of A1/C/D (n = 17), whereas in Thailand, most participants showed CRF01_AE *env* (n = 22) along with subtype B (n = 2) and CRF01_AE/B/C recombinants (n = 2). Broad and non-broad neutralizers were proportionately represented across the most sampled subtypes (Fig 1). There were no significant differences in peak neutralization breadth (Kruskal-Wallis test, P = 0.423) or breadth at three years (Kruskal-Wallis test, P = 0.966) across subtypes (Fig 1A). Among these RV217 participants, at peak neutralization breadth, 24% of participants neutralized >70% of the panel and 54% neutralized <35%, while in the subset of individuals sampled at three years post-diagnosis 38% and 50% neutralized >70% and <35%, respectively. Among the RV144 placebo participants, 18% of participants developed breadth > 70% and 42% were < 35% at three years post-diagnosis (Fig 1B).

We next quantified how much of the variation in neutralization breadth was due to transmissible viral genetics, i.e. the heritability of neutralization breadth. Using founder *env* sequences for RV217 participants and placebo recipients in the RV144 vaccine efficacy trial cohort, there was no evidence of heritability of neutralization breadth. We analyzed reconstructed phylogenies using a sequence representative of earliest-sampled sequences for each participant and a single inferred breakpoint (site 538, Δ AIC = 2145). The representative sequence for each individual could be a consensus for all earliest-sampled sequences or a consensus for each of two founder variants for infections with multiple variants. Permutations of these consensus sequences resulted in five separate alignments, each of which had a single inferred breakpoint (delta AIC>2080) and therefore ten phylogenies were analyzed. Results are reported for analyses across all reconstructed phylogenies. First, phylogenetic signal was low (Pagel’s λ = 7e-5±3.4e-6, S2A Fig). Second, neutralization breadth data were better fit by an Ornstein-Uhlenbeck (OU) than Brownian motion (BM) process (ΔAIC = 18.16±3.86), suggesting an evolutionary trend towards an optimum rather than a distribution guided strictly by phylogenetic relatedness (Fig 1C and S2B Fig). Third, the Ornstein-Uhlenbeck phylogenetic mixed model estimate converged on low heritability, *H*^*2*^ = 0.13±0.027 (HPD = 3.03e-5, 0.70; GR = 1.005) for the conditioned runs, with *α* = 15.83 (0.11, 59.30), long-term neutralization breadth mean = 40.16 (25.47, 48.42), and *σ* = 2.88 (0.38, 5.61) (Fig 1D and S2C Fig). Heritability estimates were also low when only RV144 participants with neutralization breadth measured at three years (*H*^*2*^ = 0.15) were included. Support for heritability of breadth did not increase when only non-broad and broad neutralizers were included in the above analyses (ΔAIC = 19.08, *H*^*2*^ = 0.10), nor when phylogenies were constructed using only surface sites (ΔAIC = 23.15, *H*^*2*^ = 0.10) or Ab contact sites (ΔAIC = 12.99, *H*^*2*^ = 0.04). To assess the reliability of model inferences from our data, we simulated breadth values using the same sample size (n = 126) on the consensus-sequence phylogeny under BM, OU, and stochastic models. We found that for the BM and OU models the rate of recovery for false negatives (i.e., the correct model was not supported) was 11% and that the rate of recovery for false positives (i.e., the incorrect model was supported) was 13% (S3A Fig). Data simulated under a BM model returned high estimates of *H*^*2*^ for all trees (*H*^*2*^ ≥ 0.91, S3B Fig) and data simulated on a stochastic model showed low estimates (*H*^*2*^ ≤ 0.21, S3C Fig), as expected.

### Neutralization breadth associated with infections with multiple founders

**Fig 2:**
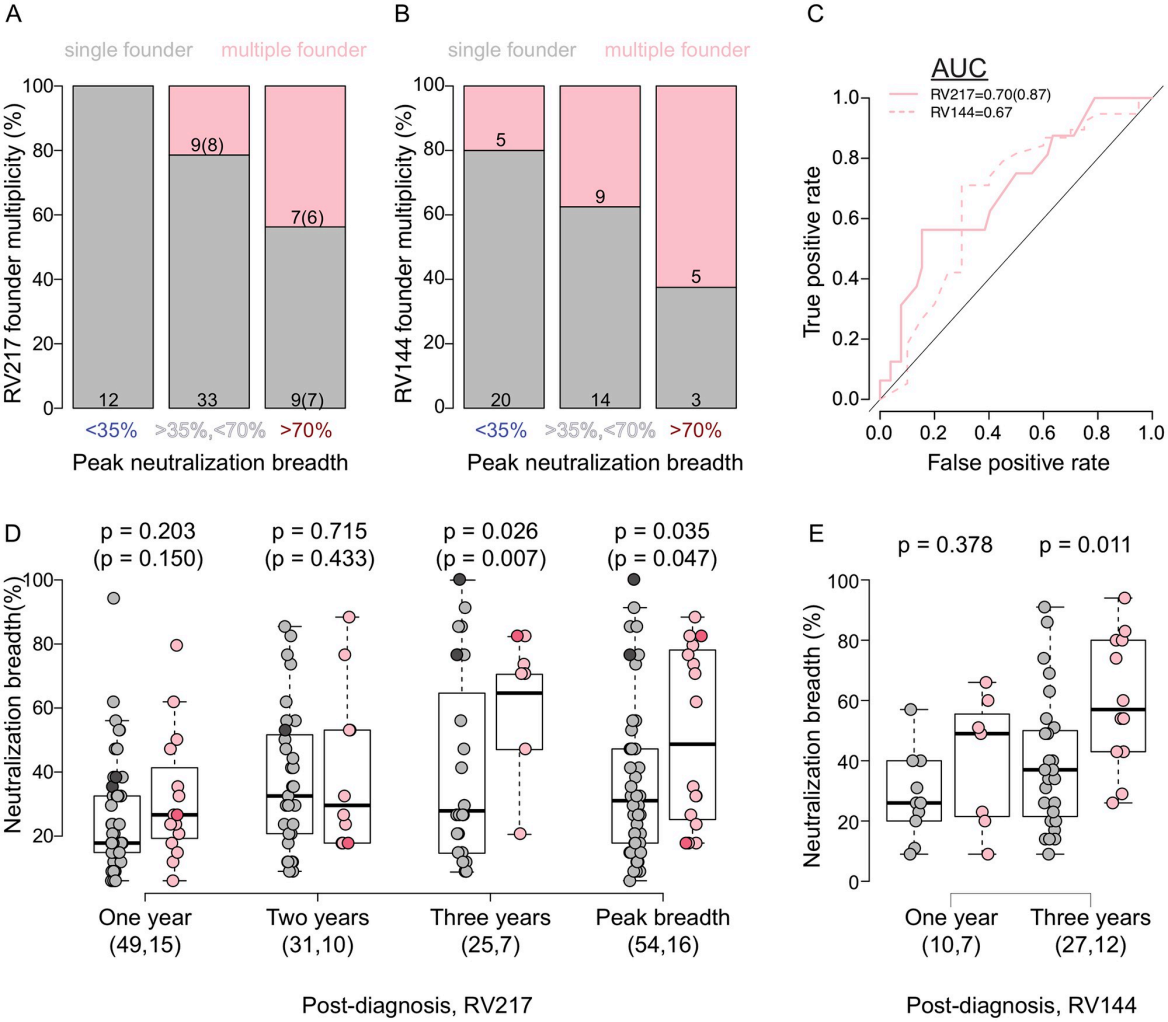
Association between infections with multiple founders and neutralization breadth. Comparisons of individuals who neutralized <35% of a 34-virus panel three to four years post-infection (non-broad neutralizers) and >70% of the panel (broad neutralizers) and had single (grey fill) or multiple (pink fill) founder infections. Individuals with neutralization estimates sampled less than two years post-infection are categorized as intermediate neutralizers (35% < neutralization breadth < 70%). Percentage of (A) RV217 participants and (B) RV144 placebo recipients within each breadth category who had infections with single (grey) and multiple (pink) founders. (C) ROC graph of the performance of founder multiplicity (pink line) for predicting neutralization breadth in RV217 participants (solid pink line) and RV144 placebo recipients (dashed pink line). The null expectation is shown as a solid black line. AUC estimates are shown. (D) Neutralization breadth in RV217 participants with single (grey) or multiple (pink) founder infections sampled at one year, two years, and three years post-diagnosis, and at peak breadth. Participants with superinfections are shown in darker shades. (E) Neutralization breadth in RV144 placebo recipients sampled at one year and three years post-diagnosis. (D,E) The number of individuals in each group is shown in parenthesis and p-values for pairwise comparisons are shown above each pair. (A,C,D) Parenthetical numbers indicate values after removing individuals identified as superinfected.

Acute infections are typically described as being established with single or multiple founder variants [19]. Inspection of sequence alignments, phylogenetic trees, and diversity metrics to evaluate the number of shared polymorphisms were used to categorize infections in the RV217 [37,38] and RV144 [40,41] cohorts. Across RV217 participants, 23% (16/70) of infections were established with multiple founder variants. The mean of median pairwise diversity in infections with single founders was 1.78 (min = 0, max = 7) character differences versus 15.10 (1,48) in infections with multiple founders at < 42 days post-diagnosis. There was similar disparity between broad neutralizers with single founders (1,0,4) versus with multiple founders (9,1,48). Participants infected with multiple founders were more likely to develop neutralization breadth than those with single founders: 44% of the participants who developed bnAbs presented multiple founders as opposed to none of the non-broad neutralizers with <35% breadth (Fisher’s exact test, p = 0.010) (Fig 2A). The other individuals with multiple founders were in an intermediate category as they developed >35% neutralization breadth but did not exceed 70%, noting that the latest samples available for sequences and neutralization breadth measurements were collected prior to year 2. Among RV144 placebo recipients, five of the eight broad neutralizers presented multiple founders as opposed to five of the 25 non-broad neutralizers (Fisher’s exact test, p = 0.036) (Fig 2B). In RV217, founder multiplicity had moderate predictive ability of breadth based on a receiver operating characteristic curve (AUC = 0.70), which was slightly lower in RV144 (AUC = 0.63) (Fig 2C). Precision recall curves, which can better account for imbalance in the distribution of neutralization breadth across participants, slightly reduced AUC estimates for RV217 (0.67) and RV144 (0.61). Neutralization breadth was significantly higher among RV217 participants infected with multiple founders compared to those with single founders at three years post diagnosis (64.71% versus 29.41%, p = 0.026) and at peak breadth (48.53% versus 32.35%, p = 0.035); the difference between infections with single and multiple founders remained significant when the individuals who later became superinfected were excluded (p = 0.007 and p = 0.047 at three years and peak breadth, respectively) (Fig 2D). There were no significant differences in neutralization breadth between broad neutralizers with single and multiple founders (S4 Fig). Among RV144 placebo recipients, breadth was also significantly higher in infections with multiple founders than single founders at three years (60% versus 39.2%, p = 0.011) (Fig 2E).

### Multiple founders boosted diversification in broad neutralizers

**Fig 3:**
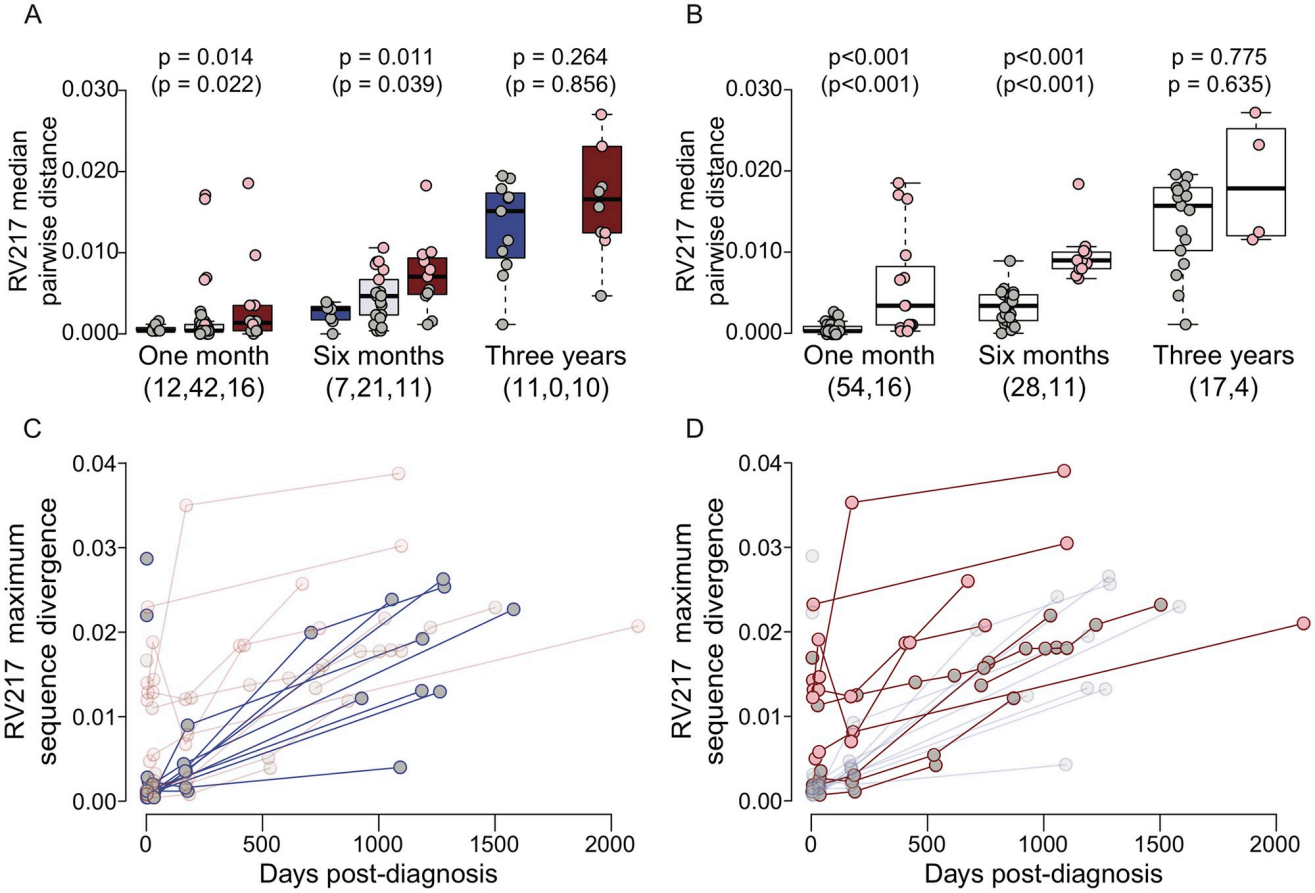
Higher diversity in infections with multiple founders in early infection. Comparisons of sequence diversity as a function of neutralization breadth and founder multiplicity in RV217 participants. Non-broad neutralizers (indicated in blue) are defined as individuals who neutralized <35% of a 34-virus panel three to four years post-diagnosis and broad neutralizers (indicated in red) as individuals who neutralized >70% of the panel. Individuals sampled less than two years post-infection (intermediate neutralizers) are also shown (indicated in lavender). Individuals with single founder infections are shown in grey and with multiple founder infections in pink. Median pairwise distance at one month, six months, and three years post-diagnosis in (A) non-broad, intermediate, and broad neutralizers and in (B) participants with single or multiple founder infections. The number of individuals in each group is shown in parenthesis and p-values for pairwise comparisons are shown above each pair. (C,D) Maximum sequence divergence over time from each participant’s consensus at the earliest sampling day for (C) non-broad neutralizers and (D) broad neutralizers. Circles indicate participants with single (grey) or multiple (pink) founder infections and lines indicate whether the individual achieved broad neutralization (red) or not (blue). A low-transparency representation of (D) is shown in (C) and vice versa. (A,B) P-values calculated after removing individuals identified as superinfected are shown in parentheses.

Median pairwise distances across sequences were significantly higher in broad neutralizers when compared to non-broad neutralizers about one month (Mann-Whitney U test, p = 0.014) and six months post-diagnosis (Mann-Whitney U test, p = 0.011), but not three years post-diagnosis (Mann-Whitney U test, p = 0.264) (Fig 3A). Similar profiles were seen when median numbers of variable sites per individual were considered (either polymorphic sites or the subset of polymorphic sites that show shared mutations and are called informative sites), as well as when analyzing minority variants at polymorphic sites (S2 and S4 Tables and S5 and S6 Figs). The diversity differences between broad and non-broad neutralizers were driven by infections with multiple founders as they had significantly higher median pairwise distances than infections with single founders at one month (0.53% versus 0.007%, p = 6.85e-5) and six months (0.87% versus 0.03%, p = 1.07e-7), but not three years (1.7% v 1.5%, p = 0.775) post-diagnosis (Fig 3B); and there were no significant differences when only infections with single founders were considered (p > 0.321). We additionally used two models to account for intra-participant diversity. First, we directly compared the spectral densities of distance matrices of each participant with the Kullback-Liebler divergence and used an analysis of similarity to determine the effect of neutralization and founder multiplicity on the resulting distance matrix. We found that there was a significant effect of neutralization breadth (p = 0.047) and founder multiplicity (p = 0.009) at one month; and of founder multiplicity at six months (p = 0.043) and three years (p = 0.019). Second, we used a linear mixed-effects model with pairwise distance as a response variable, broad- and non-broad neutralization and founder multiplicity as fixed effects, and participant as a random effect. At one month, we found that this model had decent explanatory power (R^2^ = 0.15) and significant effects from both neutralization (beta = 0.078, p < 0.001) and founder multiplicity (0.44, p < 0.001); at six months, the explanatory power was lower (R^2^ = 0.09), but maintained significant effects from neutralization breadth (0.09, p < 0.001) and founder multiplicity (0.39, p < 0.001); and at three years, the explanatory power had disappeared (R^2^ < 0.01).

In addition, we calculated the divergence from the founder consensus, i.e. the distance between the consensus representing all founder sequences and sequences sampled at different visits (Fig 3C). *Env* nucleotide sequences had diverged from the founder more in those with bnAbs than in those who did not develop bnAbs at one month (mean of medians = 0.007 versus 0.002, p = 0.050) and at six months (0.009 versus 0.004, p = 0.046), but this difference diminished at three years (0.022 versus 0.018, p = 0.080). These differences remained when superinfected individuals were removed (one month, p = 0.013; six months, p = 0.048; three years, p = 0.138). Overall, there was a modest relationship between the number of polymorphic sites per individual at about one month, six months, and three years post-diagnosis and peak neutralization breadth (*R*^*2*^ > 0.16, p < 0.038). However, this significant relationship disappeared at one month when intermediate individuals were included (*R*^*2*^ = 0.057, p = 0.051) (S7 Fig).

### Higher epitope diversity in acute infection in future broad neutralizers

**Fig 4:**
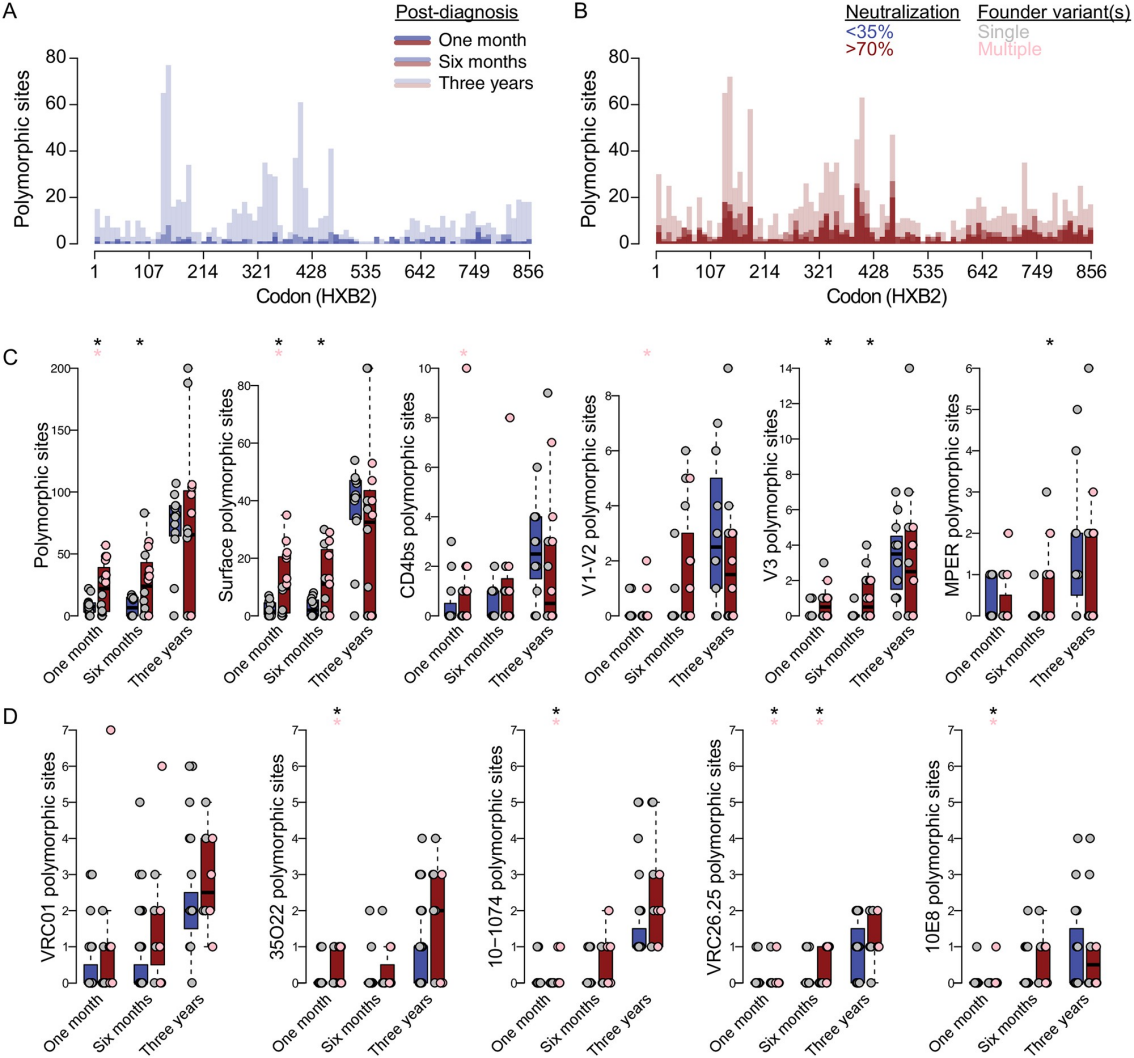
Multiple founders boosted diversification in broad neutralizers. Comparisons of polymorphic sites in RV217 participants who neutralized <35% (indicated in blue) or >70% (indicated in red) of a 34-virus panel three to four years post-diagnosis. Histogram of polymorphic sites across (A) non-broad neutralizers or (B) broad neutralizers. Boxplots of the number of polymorphic sites in non-broad (blue) and broad (red) neutralizers at one month, six months, and three years post-diagnosis, shown separately for (C) all Env sites, surface sites, CD4bs, V1-V2, V3, and MPER Ab contact sites and for (D) VRC01, 35O22, 10–1074, VRC26.25, and 10E8 epitopes. Points are shown separately for individuals infected with a single founder (grey) versus multiple founders (pink). (D) Asterisks denote significant pairwise comparisons (p ≤ 0.05) of (C) Mann-Whitney U tests and (D) Fisher’s exact test between broad and non-broad neutralizers (black) and single- versus multiple-founder broad neutralizers (pink).

We evaluated whether specific Env sites appeared to be evolving across multiple individuals. The number of sites with at least one polymorphism (i.e., polymorphic site) across individuals was three times higher in broad than non-broad neutralizers at one and six months post-diagnosis, but was only ∼20% higher at three years (Fig 4A and 4B and Table 1). Similarly, the median number of polymorphic sites per individual was significantly higher in broad compared to non-broad neutralizers at one month (median = 7.5 v 22, p = 0.013) and six months (6.5 v 24, p = 0.014) post-diagnosis, as was the subset of polymorphic sites corresponding to Env surface sites and V3 Ab contact sites (p < 0.01) (Fig 4C and Table 1). Among broad-neutralizers, at one month post-diagnosis, the number of polymorphic sites per individual was significantly higher in individuals with multi-founder infections compared to those with single founders for all Env sites (p = 0.001), surface sites (p = 0.001), CD4bs (p = 0.007) and V1-V2 Ab contact sites (p = 0.022) (Fig 4C).

**Table 1:** Number of polymorphic sites in broad and non-broad neutralizers. Median numbers are listed for all Env sites and different subsets at different times after HIV-1 diagnosis in. individuals who developed. bnAbs or not.

	Time	Total	Surface	CD4Bs	V1V2-glycan	V3-glycan	MPER
**Broad**	**1 month**	22	9.5	0	0	0.5	0
	**6 months**	24	11	1	0	0.5	0
	**3 years**	65.5	32.5	0.5	1.5	2.5	0
**Non-broad**	**1 month**	7.5	1.5	0	0	0	0
	**6 months**	6.5	2	0	0	0	0
	**3 years**	81	41.5	2.5	2.5	3.5	1

We compared the epitope sequence for five representative antibodies targeting the CD4bs (VRC01), gp120/gp41 interface (35O22), V3-glycan (10–1074), V2 apex (VRC26.25) and MPER (10E8). We found a higher epitope diversity (number of polymorphic sites) at one month in broad neutralizers compared to non-broad neutralizers and between infections with multiple founders compared to single founders in 35O22, 10–1074, VRC26.25 and 10E8 (Fisher’s exact test, p ≤ 0.05). Among broad neutralizers, epitope diversity was higher in infections with multiple founders vs. single founders in 35O22, 10–1074, VRC26.25 and 10E8 (p ≤ 0.05). These differences only persisted to six months in VRC26.25 and all differences disappeared at three years (Fig 4D).

Finally, to understand the relationship between initial diversity and the development of breadth, we used a generative additive model on broad and non-broad neutralizers at one month post-diagnosis. We found that the number of polymorphic sites was significantly predictive of peak neutralization breadth mean (p = 0.006) and standard deviation (p = 0.0002). Based on this, the model predicted that each polymorphic site added 0.35% breadth. When broad neutralizers with a single founder infection were removed from the analysis, the predicted increase per polymorphic site was slightly higher at 0.5%.

## Discussion

We analyzed HIV-1 *env* sequences sampled in acute infection and through three to four years in a diverse cohort of antiretroviral naïve individuals. In a prior study, we showed that 16 of 70 individuals (23%) developed broadly neutralizing antibodies (broad neutralizers defined as those whose plasma neutralized >70% of a panel of 34 viruses) [26]. By comparing >1,000 sequences sampled in acute HIV-1 infection to the subsequent breadth these individuals elicited, we showed that HIV-1 diversity in acute infection is associated with bnAb development. We demonstrated that the quarter of individuals who were infected with multiple founder variants were over-represented three years later among those who developed broadly neutralizing responses.

First, we tested whether sequences that shared genetic attributes yielded similar levels of bnAbs. Our cohort was constituted by infections from different subtypes; however, comparisons across subtypes were limited given the few individuals in each subtype category, possibly explaining the lack of significant differences observed. To further examine whether the relatedness between specific sequences may promote the development of bnAbs, we evaluated the heritability of breadth but found no such evidence. A study by Kouyos and colleagues in 303 pairs of individuals with phylogenetically linked viruses found a weak but significant association between neutralization characteristics in these linked pairs (on average, Spearman ρ = 0.11, p < 0.001) [43]. They showed that about 13% of the variability seen in the neutralization response was attributed to viral determinants. It is possible that our cohort was too small to illustrate this effect, as their study took advantage of a large cross-sectional cohort of chronically infected individuals (n = 4,281). However, the differing results could also be due to our implementation of a process-based phylogenetic analysis as opposed to their usage of correlations between transmission pairs, as pairwise tests of evolutionary correlation we employed were shown to have high type-I error [44].

Second, we showed that infections that were established with multiple founders were more likely to lead to the development of bnAbs. Almost half of the individuals who developed bnAbs in the RV217 cohort had initially been infected by multiple founder variants. We showed that this association between multiple founders and the development of bnAbs was robust to the exclusion of the three participants with multiple founders who were identified as super-infected one to two years later. Since the development of bnAbs in these participants could be linked to the superinfection as previously reported, we excluded these participants, yet p-values remained similar [28–34]. Importantly, the analysis of the HIV-1 diagnosis sequence and neutralization breadth data in the RV144 cohort reproduced the positive association between founder multiplicity and development of bnAbs seen in the RV217 cohort. Among RV144 study participants who became infected, only placebo participants developed neutralization breadth [42] and these placebo participants showed higher *env* diversification in early infection than vaccine recipients [41]. Logically, we found that higher *env* diversity in acute infection was associated with an increase in neutralization breadth when compared to the more homogeneous infections. This relationship was driven by infections with multiple founders as these infections showed higher diversity than those with single founder infections. Infections with multiple founders were also correlated with higher divergence from the founder sequence which associated with bnAbs. Nonetheless, one limitation of our study is that we analyzed a small number of genomes per participant and the lack of deep-sequencing data constrains our ability to capture the viral population diversity. While we showed an association between *env* diversity during acute infection and development of bnAbs years later, we also found that certain individuals with a single founder infection and limited initial diversity were also able to develop bnAbs, indicating that early *env* diversity is not the only route to develop breadth. We must also allow for the possibility that early *env* diversity interacts indirectly with the development of breadth via a cofactor, which we did not identify.

We showed that each additional polymorphism during acute infection resulted, on average, in about a half of a percentage increase in peak neutralization breadth. Because achieving neutralization breadth depends on the induction of multiple antibody specificities, we tested whether the relationship between early diversity and later neutralization was visible at the epitope level. Indeed, individuals who later developed bnAbs had more diverse epitopes for a set of representative bnAbs when compared to individuals who did not develop bnAbs. The link between Env diversity and neutralization breadth is not novel. The process of antibody escape (which can promote the maturation of bnAbs) as a driver of HIV-1 diversification has long been recognized [3,21,45–47]. In turn, HIV-1 diversity has been associated with neutralization breadth, both during early [24] or chronic infection [25]. One unique aspect of our study is that we sequenced infections in the first days after viremia became detectable (allowing us to evaluate the effect of the multiplicity of founder variants) and longitudinally. Importantly, we showed that the association between Env diversity and later neutralization breadth was identified in the first month after diagnosis, diminished at six months after infection and had disappeared at two or three years after infection.

Taken together, these data suggest a novel vaccine strategy incorporating minimally diverse antigens to promote the elicitation of bnAbs. The goal would be to design a vaccine constituted by minimally distant Env sequences that reflect the diversity found in infections with multiple founders. Leading vaccine strategies are based on lessons from natural infections. Germline targeting strategies seek to improve the longitudinal process seen in individuals infected with single HIV-1 founder variants who later developed breadth [48–50]. The vaccine antigens seek to reproduce the directional process that leads to breadth in a minority of individuals by using antigens that correspond to stepwise stages of the co-evolution between the virus and the neutralizing response. In contrast, the variability-inclusive strategies, such as the mosaic approach, are reminiscent of the diversity seen in superinfections [51–54]. As such, mosaic antigens are designed to be maximally distant to cover a large fraction of circulating viruses, usually to span multiple subtypes. Cases of superinfections that led to breadth support that immunizations with mosaic-like diverse viral strains could lead to the development of antibody responses against these distant viruses thereby potentiating cross-reactive responses.

Our findings lead us to propose a novel vaccine strategy employing a set of minimally distant antigens that would match the diversity seen in infections with multiple founders, i.e. differing by less than 1%. We hypothesize that an initial immunization with minimally distant antigens would initiate a more synergistic and coordinated initial response targeting slightly divergent epitopes by kicking off a back-and-forth process. While we surmise that priming with a set of related antigens that include variation at antibody contact sites could shorten the path towards bnAbs, we recognize that priming with a set of selected founder variants does not fully recapitulate the constantly changing landscape of variants and recombinants thereof observed over time in an acute infection established with multiple lineages. Nonetheless, exposure to distinct but related antigen variants could permit a more cross-reactive affinity maturation process—enabling a longer toggle towards optimal clones in the germinal centers. Additional work on this cohort supports the important role of the initial stages of infection as participants who later developed broadly neutralization breadth were distinguished from other participants by the higher capacity of their naïve B cells to engage founder Env in the first months of infection [26].

The potential advantage of using a set of related variants as immunogens suggested by our findings is supported by other relevant work. Sheward and colleagues demonstrated that superinfection did not broaden the initial antibody response but rather added new antibody responses, hence resulting in an additive process of neutralization breadth expansion [55]. Similarly, animal studies that tested different combinations of SOSIP trimers showed that antigenically distinct trimers corresponding to different subtypes elicited independent autologous neutralizing responses rather than synergistic ones [56,57]. The above studies suggest that the additive process seen with superinfections or immunization with distinct SOSIPs could correspond to the activation of independent germinal centers by distinct HIV-1 antigens. Whether using closely related, minimally distinct variants in a vaccination strategy could redirect the antibody development towards a synergistic cross-reactive process remains to be evaluated.

Nonetheless, the above studies together with our findings that infections with multiple founder variants promoted the development of neutralization breadth indicate that using slightly variant antigens (similar to those found in multi-founder infections) may propel vaccination strategies towards the elicitation of bnAbs.

## Materials and methods

### Ethics statement

All participants signed written informed consent and participated in protocols approved by local (either Kenyan, Tanzanian, Ugandan or Thai) and US (Walter Reed Army Institute of Research) Institute Review Boards. The investigators have adhered to the policies for protection of human subjects as prescribed in AR 70–25.

### Study design

This study included people living with HIV-1 who were enrolled in the RV217 prospective cohort [35] and the RV144 vaccine efficacy trial (NCT00337181) [58]. The RV217 cohort enrolled 3,173 seronegative individuals at increased risk for HIV-1 acquisition in four countries (Kenya, Tanzania, Thailand and Uganda). Participants were tested with an HIV-1 RNA test twice-weekly, leading to 155 acute HIV-1 infection diagnoses. RV144 trial participants were tested for HIV-1 infection every six months and 110 participants became infected with CRF01_AE viruses (66 vaccine and 44 placebo). Participants were followed for up to five years and all samples were obtained prior to anti-retroviral treatment initiation. Plasma samples were used for sequencing HIV-1 [37,38,40,41] and measuring neutralization breadth [26,42] using the same methodology.

### Sequence alignments

Nucleotide sequences were restricted to the *env* region using Gene Cutter (www.hiv.lanl.gov/content/sequence/GENE_CUTTER/cutter.html). Sequences were aligned separately for each individual using MAFFT v7.419 [59] and edited manually with AliView.[60]. Evidence of hypermutations within individual alignments was determined with Hypermut [61] and all hypermutated sequences were removed. The nucleotide sequences were then translated. Before translation, ambiguous sites were replaced by consensus nucleotides (as per the IUPAC ambiguity code). Following translation, sequences with frame shifts or stop codons outside the C-terminus were removed. The resulting set of amino acid sequences were then aligned to the reference group M sequences [11]. Hypervariable loops were evenly split to each end in the alignment and sequences with long singleton deletions (≥ 3 AA) in conserved regions were removed.

Env sites that were considered accessible to antibodies were defined based on the resolved closed prefusion Env trimer structure (PDB code 5FYJ) [62]as those with a relative accessible surface area (ASA) [63] greater than 0.08, as described in [64]. The relative ASA was calculated as the ratio of the side chain ASA (scASA, where *C*_*α*_ are included) over the maximum ASA (maxASA) of a residue [65]. The residue depth, which corresponds to the distance from the residue to the solvent, was calculated using the DEPTH webserver (http://cospi.iiserpune.ac.in/depth/) [66]. Two depth definitions were used to exclude surface sites in cavities of different sizes. For large cavities, such as the center of the closed prefusion trimer, the parameter ‘neighborhood waters’ was set to its maximum value (= 5) to calculate the depth and surface sites with depth_5HOH_ > 8 *Å* were excluded. For small cavities, the parameter ‘neighborhood waters’ was set to its default value (= 2) to calculate the depth and surface sites with depth_2HOH_ > 4.5 *Å* were excluded.

### Heritability of neutralization breadth

Majority consensus sequences from the first sampling were computed using amino acid sequences and aligned with a profile hidden Markov model [67] for each of the RV217 participants (n = 70) and each of the placebo recipients in the RV144 vaccine efficacy trial cohort (n = 58) with sequences and neutralization breadth data. Two of the RV144 placebo recipients were removed (AA057, AA117) due to having outlying dates for breadth samples. Population-level alignments were constructed using the complete *env* sequences with consensus sequences for each participant and consensus sequences for each founder (for up to two founders) in participants that were infected with multiple founder variants. This resulted in five alignments with consensus sequences from: (i) each participant in RV217 and RV144; (ii) founder1 in RV217 and RV144; (iii) founder 1 in RV217 and founder 2 in RV144; (iv) founder 2 in RV217 and RV144; and (v) founder 2 in RV217 and founder1 in RV144. Evidence of recombination breakpoints was assessed in each alignment using GARD. Phylogenies were constructed using IQ-Tree [68] and ModelFinder [69] for all individuals using the complete *env* sequences, taking account of detected breakpoints. Separate analyses were run only using RV144 placebo recipients with neutralization breadth sampled at one year (n = 17) or at three years (n = 39) post-diagnosis. Neutralization breadth was ln-transformed for phylogenetic analyses. Heritability, H^2^, of neutralization breadth was computed based on (i) pairwise sequence similarity, (ii) maximum-likelihood fits of trait evolution models, and (iii) phylogenetic mixed model (PMM) analyses. (i) Pairwise sequence similarity was estimated using the identity matrix for each pair of consensus sequences sampled one month post-diagnosis. Trait differences were measured as the absolute difference in neutralization breadth between pairs. *H*^2^, which ranges from no heritability (0) to high heritability (1), was then measured as the coefficient of determination, $R^{2}=1-\frac{\sigma (x-G)}{\sigma^{2}(x)}$, where *σ*^2^ is the sample variance, *x* is the measured neutralization breadth, and *G* is the estimated sequence similarity. (ii) Assigning peak neutralization breadth to each tip, phylogenetic signal was estimated using Pagel’s λ [70] and univariate multiple rates models were fit to the phylogeny and neutralization breadth data under Brownian motion (BM), Ornstein-Uhlenbeck (OU), and early burst (EB) models of evolution using mvMORPH [71]. Model fits were compared with Akaike weights [72]. (iii) For PMM analyses, the trait value at time t, (*t*), is equal to the heritable component at time *t*, (*t*), plus an environmental component, *e*. A BM PMM assumes that (*t*) evolves according to a stochastic process defined by sample variance, *σ*, and variance due to an environmental component, *σ*_*e*_. An OU PMM assumes that (*t*) follows a random walk that trends towards some optimum value, where the strength of the trend is determined by an attractor, *α*. In both cases, (*t*) is a function of model parameters, which can be inferred by estimating the log-likelihood of the data on the phylogeny. Based on parameter estimates inferred on fits of each model, *H*^2^ can be calculated as by:

$$H_{BM}^{2}=\frac{t\sigma^{2}}{t\sigma^{2}+\sigma_{e}^{2}}$$

and

$$H_{OU}^{2}=\frac{\sigma^{2}\left(1-e^{2\alpha t}\right)}{\sigma^{2}\left(1-e^{2\alpha t}\right)+2\alpha \sigma_{e}^{2}}$$

[73].

BM and OU PMMs were fit to the phylogeny and neutralization breadth data using maximum likelihood. Based on best-fit parameter estimates, MCMC chains were run assuming a constant likelihood and then run using Metropolis sampling [74] from the posterior distribution conditioned on the initial run. Each chain was run for 10^5^ iterations, from which the highest posterior distribution (HPD) was calculated. Gelman-Rubin statistics (GR) were used to estimate whether the runs were drawn from similar distributions, where *GR >* 1.01 suggests samples have not converged. PMM analyses were run using the R package POUMM [75].

The same analyses were run including only participants that had neutralization breadth estimated more than three years post-infection (n = 67), as well as on phylogenies constructed using only surface sites or Ab contact sites. To assess the statistical power of the maximum-likelihood and phylogenetic mixed models on our data, we simulated tip data for each phylogeny and dataset. Tip data were simulated 1000 times each under either a BM or OU [71] model or a stochastic model. False negatives and positives were assessed from the maximum-likelihood model analyses based on AICc values with the BM and OU models; and *H*^*2*^ scores were compared for each simulated dataset on the BM and stochastic models.

### Diversification analysis

We analyzed sequence diversity and divergence, phylogenetic diversity, and site-specific diversity as an effect of neutralization breadth, founder multiplicity, and time since diagnosis.

Diversity estimates for individual *env* alignments sampled at one and six months and three years post-diagnosis were measured using maximum and median pairwise distances of nucleotide sequences assuming a general time-reversible (GTR) model [76]. Divergence estimates for individual *env* alignments were estimated at each day of sampling as the maximum distance of nucleotides sequences from the individual consensus assuming a GTR model. Pairwise distances in nucleotide sequences were estimated using the R package phangorn [77].

Site-specific diversity was measured on individual amino acid alignments on all sequences sampled at one and six months and at three years post-diagnosis as mutations found in at least one sequence (polymorphic site), as mutations found in at least two sequences (informative site), and as mutations found in more than one individual at the same site (shared polymorphisms). Mutated sites were then cross-referenced with surface sites and with known and predicted antibody contact sites. Polymorphisms were computed for sequences from each participant for Env sites that corresponded to contact sites for the following antibodies: VRC01, 35O22, 10–1074, VRC26.25 and 10E8. Analyses were run separately for alignments of sequences sampled at one month, six months, and three years post-diagnosis.

### Statistical analysis

Pairwise comparisons were made using Mann-Whitney U tests. Two-sample Kolmogorov-Smirnov tests were used to compare distributions of polymorphic sites. Spearman’s *ρ* was used to estimate correlations between pairwise variables. Receiver operating characteristic (ROC) curves were computed for peak neutralization breadth and founder multiplicity. Classifier data were created from the peak neutralization breadth data and founder status. Areas under the curve (AUCs) were then calculated for each ROC. To account for imbalance in the distributions of neutralization breadth in each cohort, precision recall curves were also computed and their AUCs calculated. ROC and precision recall curves were computed with *ROCR* [78]. To account for intra-participant diversity we used an analysis of similarity and a linear mixed-effects model. For the analysis of similarity, we computed the spectral densities of distance matrices for each participant at one month, six months, or three years post-diagnosis and used either broad and non-broad neutralization or founder multiplicity as a grouping factor over 1000 permutations [79]. A linear mixed-effects model was constructed with pairwise distances at one month, six months, or three years as a response variable, broad or non-broad neutralization and founder multiplicity as fixed effects, and participant as a random effect. These were constructed using lme4 [80]. A generative additive model on broad versus non-broad neutralizers was used to predict the median addition of percentage breadth per polymorphic site during the first month of infection. This was done with the package gamlss [81].

## Supporting information

S1 Table Number of sequences sampled per participant at each time point. (CSV)

S2 Table Polymorphic sites in participants in the RV217 cohort at one month post-diagnosis. (CSV)

S3 Table Polymorphic sites in participants in the RV217 cohort at six months post-diagnosis. (CSV)

S4 Table Polymorphic sites in participants in the RV217 cohort at three years post-diagnosis. (CSV)

S1 Fig Distribution of sequences sampled over time per RV217 participant. The number of sequences sampled at each day since diagnosis is shown for each individual. The circle sizes are scaled to the number of sequences (see legend). Circles are colored according to whether the participant’s plasma neutralized <35% (indicated in blue) or >70% (indicated in red) of a 34-virus panel three to four years post-diagnosis, or neutralization breadth was sampled less than two years post-infection (lavender). (A) The number of sequences sampled per individual by day of sampling and (B) clustered into bins for one month, six months, one year, two years, and three years.
(TIF)

S2 Fig Phylogenetic heritability for phylogenies constructed from RV217 and RV144 consensus sequences. Consensus sequences were calculated from either of two founders within participants infected with multiple founder variants. Phylogenies were constructed for combinations of each founder sequence from RV217 and RV144 participants. Two phylogenies were constructed for each combination of sequences based on detection of a single recombination breakpoint. (A) Distribution of Pagel’s lambda. (B) The difference between the minimum corrected AIC score and the score for each of the fit models: Brownian motion (BM), Ornstein-Uhlenbeck (OU), and Early burst (EB). Scores are shown for two phylogenies for each set of reconstructed sequences, corresponding to a single inferred breakpoint. (C) The distribution of heritability estimates, H^2^, calculated across three runs of an OU phylogenetic mixed model.
(TIF)

S3 Fig Sensitivity and specificity of heritability estimates on simulated data. **(A)** Density plot of the difference in AICc estimates for Brownian motion (BM) and Ornstein-Uhlenbeck (OU) models fit to data simulated under a BM (grey) or OU (purple) model on the RV217 and RV144 phylogeny. For data simulated under a BM model (grey), estimates greater than zero indicate false negatives (hatched grey); for data simulated under a OU model (purple), estimates less than zero indicate a false positive (hatched purple). (B,C) The frequency of heritability scores retrieved from fitting a phylogenetic mixed model on data simulated under a BM model (B, grey) and a stochastic model (C, green) on the RV217 and RV144 phylogeny. For each model (BM, OU, and stochastic), 1000 simulations were run.
(TIF)

S4 Fig Neutralization breadth over time in broad neutralizers with single or multiple founder infections in the RV217 cohort. Boxplot of neutralization breadth sampled in infections with a single founder (grey) and multiple founders (pink) at one year, two years, or three years post-diagnosis, and at peak neutralization breadth. The number of individuals in each group is shown in parenthesis and p-values for pairwise comparisons (Mann-Whitney U test) are shown above each pair. P-values calculated after removing individuals identified as superinfected are shown in parentheses.
(TIF)

S5 Fig Higher numbers of polymorphic sites in broad neutralizers and in infections with multiple founders. (A) Polymorphic sites per individual and (B) informative sites per individual in non-broad (blue) and broad (red) neutralizers at one month, six months, and three years post-diagnosis. Informative sites are polymorphic sites where substitutions are shared in at least two sequences. The number of individuals in each group is shown in parenthesis and p-values for pairwise comparisons (Mann-Whitney U test) between non-broad and broad neutralizers and between broad neutralizers infected with a single founder (grey) or multiple founders (pink) are shown above each pair. P-values calculated after removing individuals identified as superinfected are shown in parentheses.
(TIF)

S6 Fig Minority variants at polymorphic sites in the RV217 cohort. Boxplots of the percentage of minority variants across (A) non-broad neutralizers (blue) and (B) broad neutralizers (red), (C) the maximum percentage of minority variants per polymorphic site in non-broad and broad neutralizers, and (D) the maximum percentage of minority variants across polymorphic sites per individual in non-broad and broad neutralizers at one month post-diagnosis. The same is shown for sequences sampled at (E-H) six months post-diagnosis and (I-L) three years post-diagnosis. Infections with a single founder are indicated in grey and with multiple founders in pink. P-values for pairwise comparisons (Mann-Whitney U test) between non-broad and broad neutralizers and between broad neutralizers with a single founder infection or multiple founder infections are indicated above pairs.
(TIF)

S7 Fig Relationship between the number of polymorphic sites and neutralization breadth in the RV217 cohort. Non-broad neutralizers (indicated in blue) are defined as individuals who neutralized <35% of a 34-virus panel three to four years post-diagnosis and broad neutralizers (indicated in red) as individuals who neutralized >70% of the panel. Individuals sampled less than two years post-infection (intermediate neutralizers) are also shown (indicated in lavender). Participants with a single founder infection are indicated with a grey border and with multiple founder infections with a pink border. Peak neutralization breadth as a function of the number of polymorphic sites per individual at (A) one month, (B) six months, and (C) three years post-diagnosis. Best-fit regression lines are shown for all participants (solid) and for only participants with a single founder infection (dashed). Legends provide best-fit regression statistics for all participants (solid line), only participants with a single founder infection (dashed), and only participants that were non-broad or broad neutralizers (red, blue circles); parenthetical statistics are shown for analyses without individuals with superinfections.
(TIF)
