# Supplementary figures and images for: HIV-1 infections with multiple founders associate with the development of neutralization breadth
Source: PLoS Pathog. 2022 Mar 18;18(3):e1010369. doi: 10.1371/journal.ppat.1010369 (PMC8967031; doi:10.1371/journal.ppat.1010369)

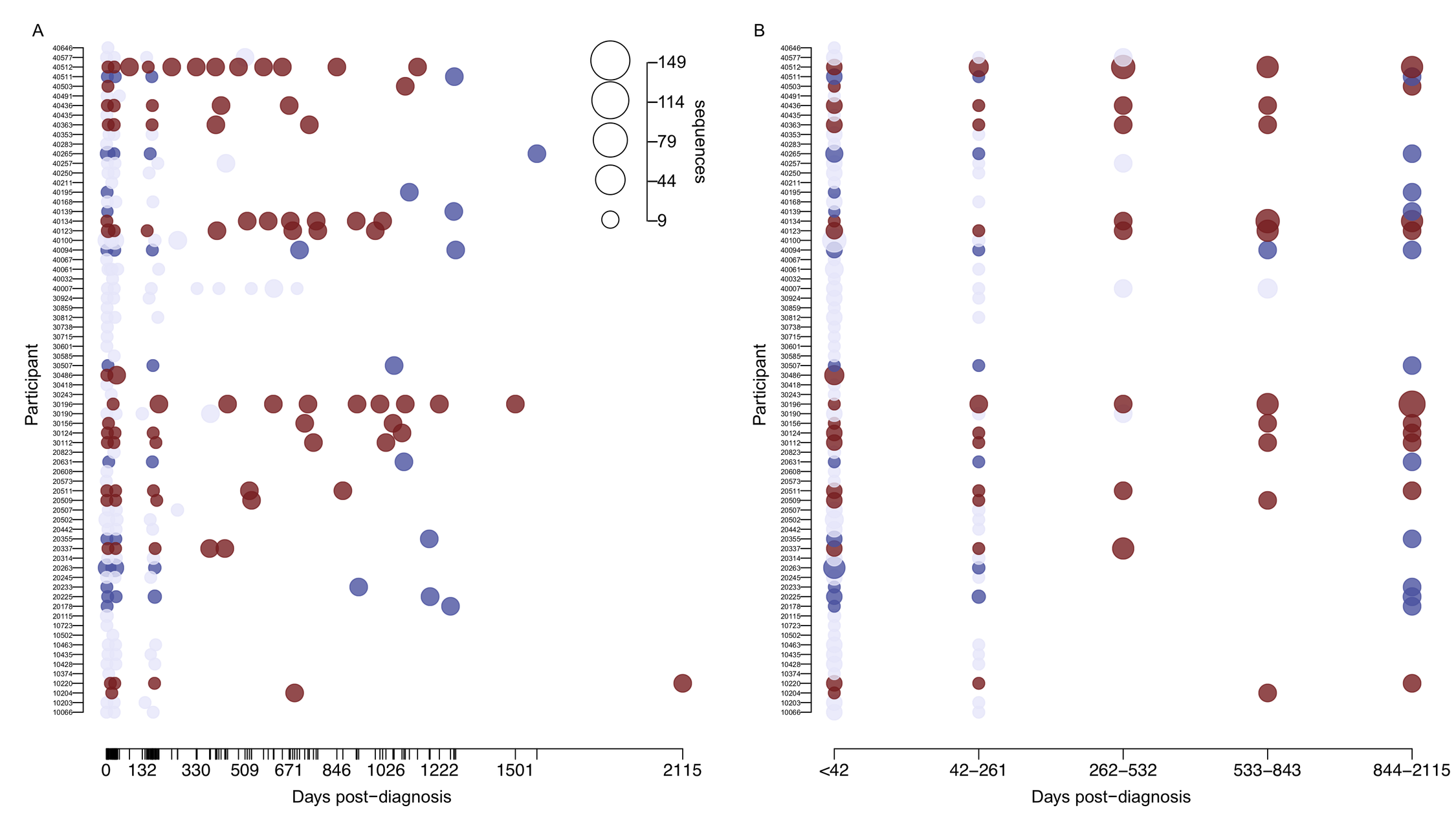

Supplement: S1 Fig — The number of sequences sampled at each day since diagnosis is shown for each individual. The circle sizes are scaled to the number of sequences (see legend). Circles are colored according to whether the participant’s plasma neutralized <35% (indicated in blue) or >70% (indicated in red) of a 34-virus panel three to four years post-diagnosis, or neutralization breadth was sampled less than two years post-infection (lavender). (A) The number of sequences sampled per individual by day of sampling and (B) clustered into bins for one month, six months, one year, two years, and three years. (TIF) [file ppat.1010369.s005.tif]

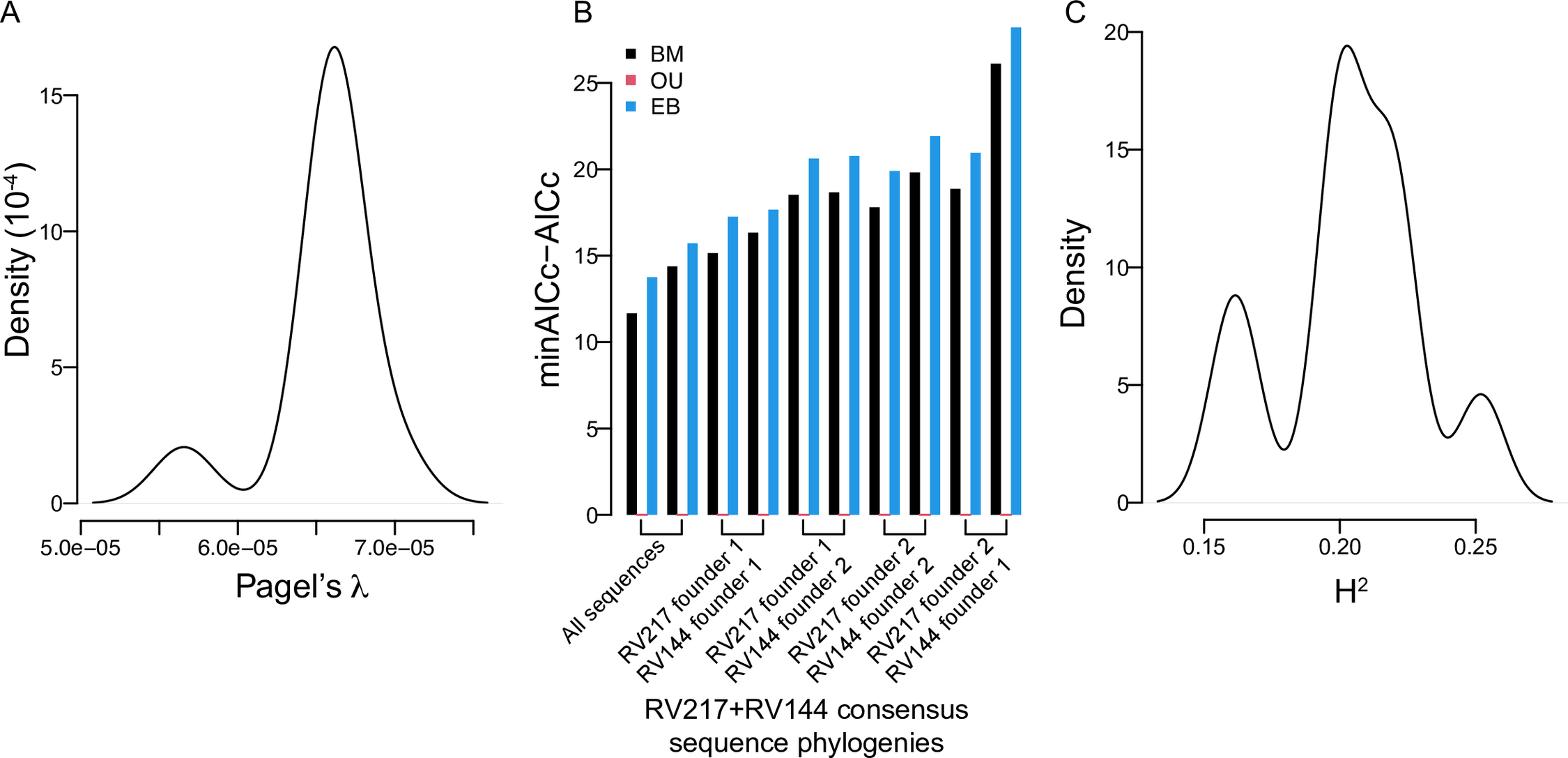

Supplement: S2 Fig — Consensus sequences were calculated from either of two founders within participants infected with multiple founder variants. Phylogenies were constructed for combinations of each founder sequence from RV217 and RV144 participants. Two phylogenies were constructed for each combination of sequences based on detection of a single recombination breakpoint. (A) Distribution of Pagel’s lambda. (B) The difference between the minimum corrected AIC score and the score for each of the fit models: Brownian motion (BM), Ornstein-Uhlenbeck (OU), and Early burst (EB). Scores are shown for two phylogenies for each set of reconstructed sequences, corresponding to a single inferred breakpoint. (C) The distribution of heritability estimates, H2, calculated across three runs of an OU phylogenetic mixed model. (TIF) [file ppat.1010369.s006.tif]

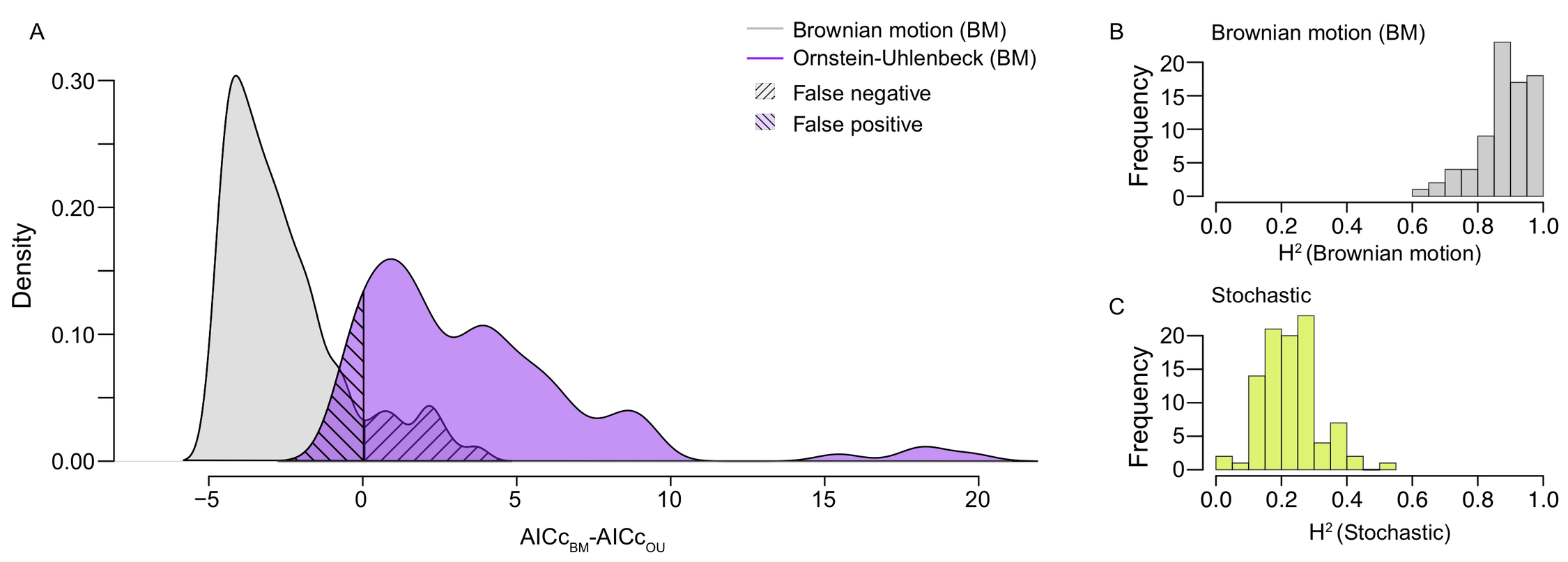

Supplement: S3 Fig — (A) Density plot of the difference in AICc estimates for Brownian motion (BM) and Ornstein-Uhlenbeck (OU) models fit to data simulated under a BM (grey) or OU (purple) model on the RV217 and RV144 phylogeny. For data simulated under a BM model (grey), estimates greater than zero indicate false negatives (hatched grey); for data simulated under a OU model (purple), estimates less than zero indicate a false positive (hatched purple). (B,C) The frequency of heritability scores retrieved from fitting a phylogenetic mixed model on data simulated under a BM model (B, grey) and a stochastic model (C, green) on the RV217 and RV144 phylogeny. For each model (BM, OU, and stochastic), 1000 simulations were run. (TIF) [file ppat.1010369.s007.tif]

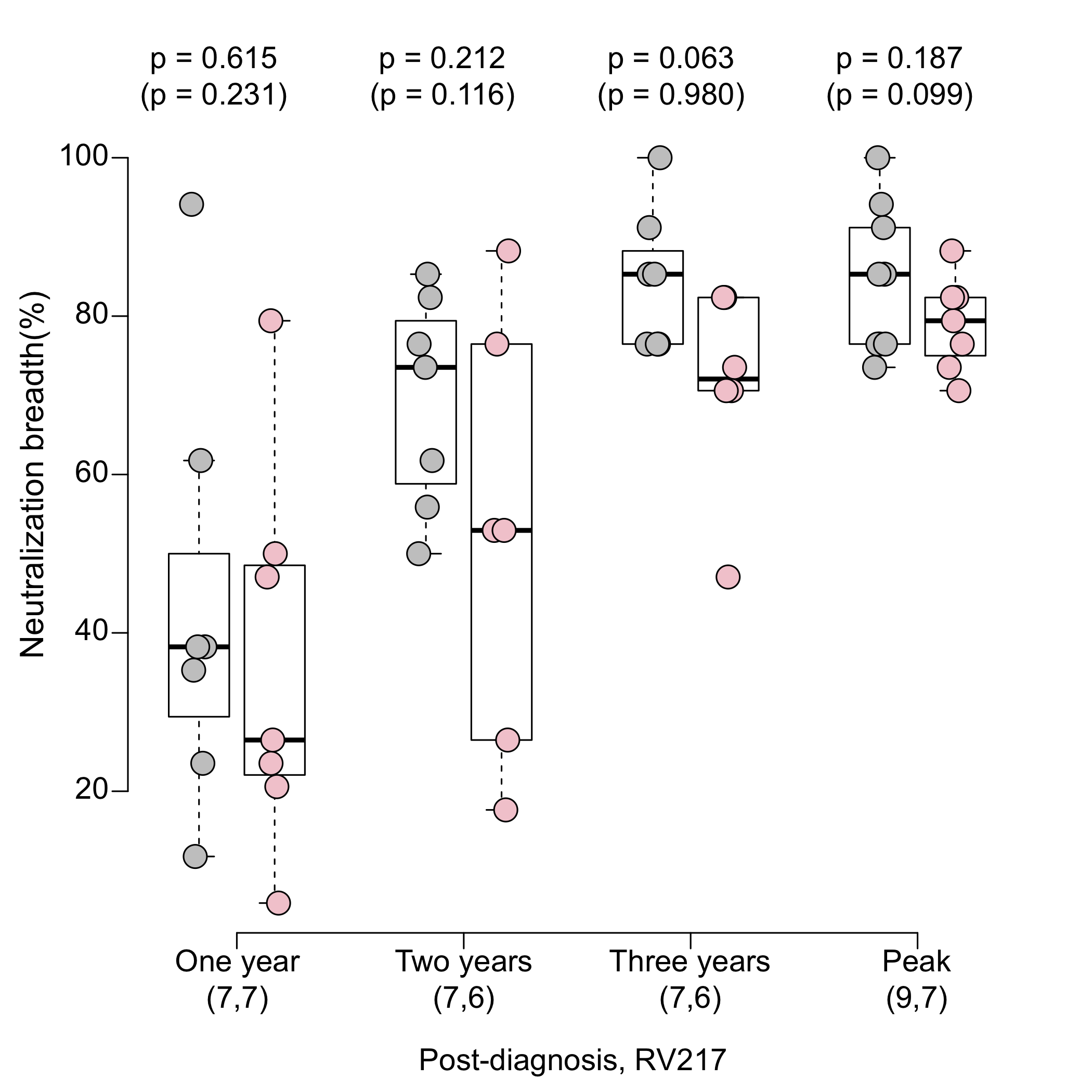

Supplement: S4 Fig — Boxplot of neutralization breadth sampled in infections with a single founder (grey) and multiple founders (pink) at one year, two years, or three years post-diagnosis, and at peak neutralization breadth. The number of individuals in each group is shown in parenthesis and p-values for pairwise comparisons (Mann-Whitney U test) are shown above each pair. P-values calculated after removing individuals identified as superinfected are shown in parentheses. (TIF) [file ppat.1010369.s008.tif]

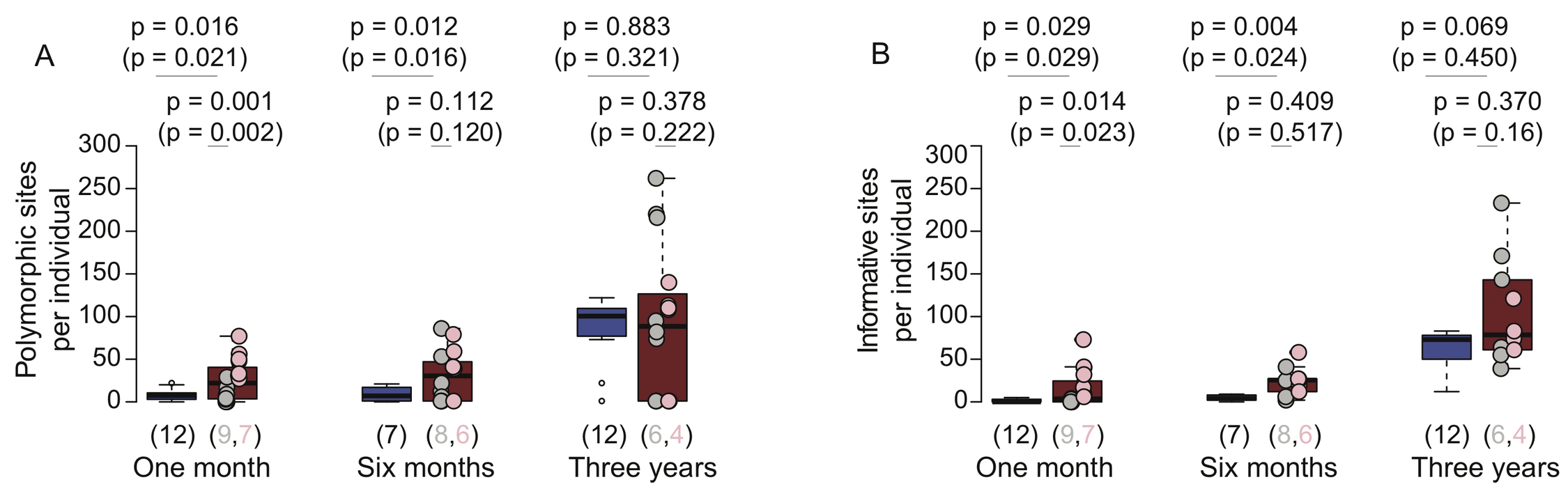

Supplement: S5 Fig — (A) Polymorphic sites per individual and (B) informative sites per individual in non-broad (blue) and broad (red) neutralizers at one month, six months, and three years post-diagnosis. Informative sites are polymorphic sites where substitutions are shared in at least two sequences. The number of individuals in each group is shown in parenthesis and p-values for pairwise comparisons (Mann-Whitney U test) between non-broad and broad neutralizers and between broad neutralizers infected with a single founder (grey) or multiple founders (pink) are shown above each pair. P-values calculated after removing individuals identified as superinfected are shown in parentheses. (TIF) [file ppat.1010369.s009.tif]

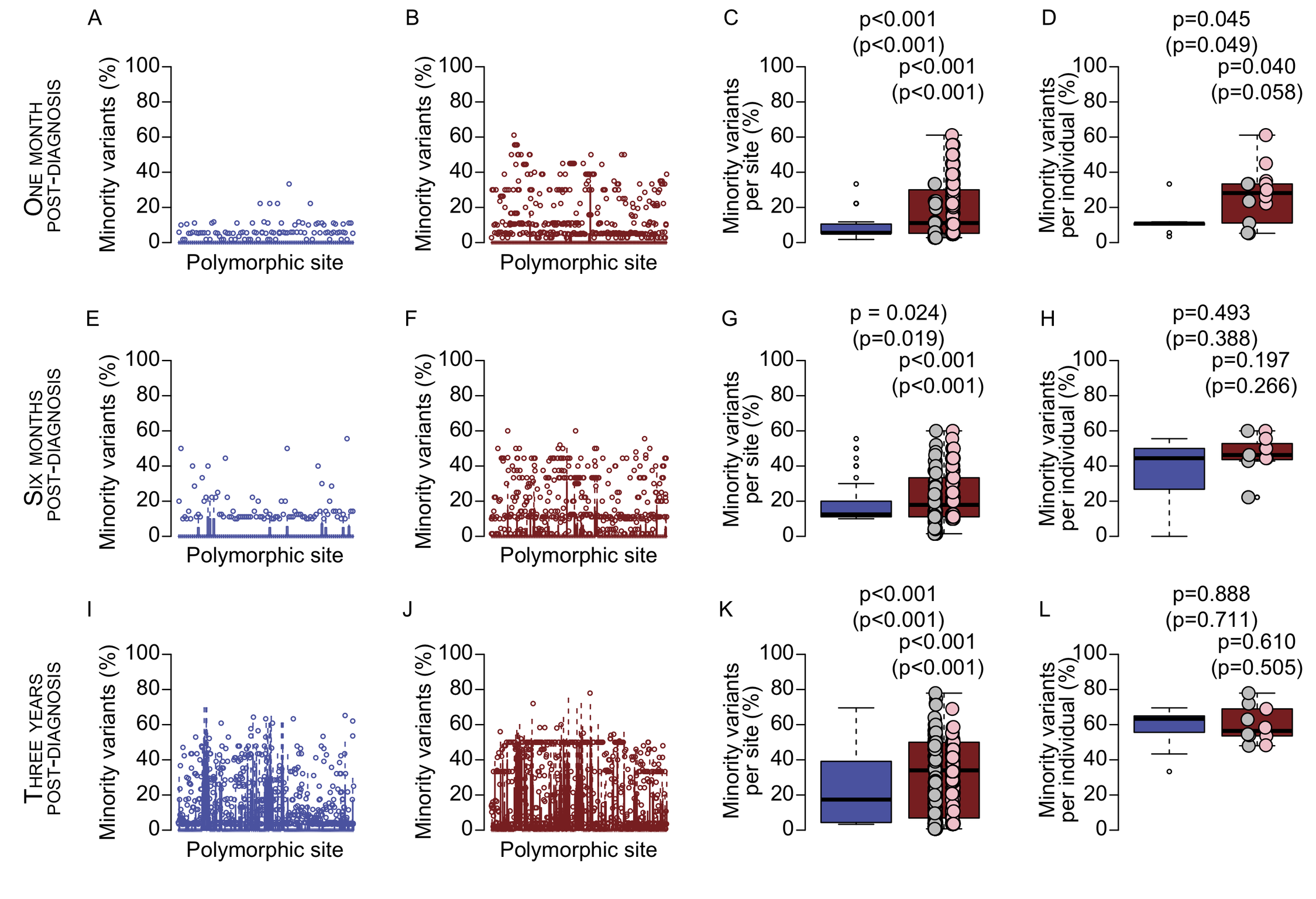

Supplement: S6 Fig — Boxplots of the percentage of minority variants across (A) non-broad neutralizers (blue) and (B) broad neutralizers (red), (C) the maximum percentage of minority variants per polymorphic site in non-broad and broad neutralizers, and (D) the maximum percentage of minority variants across polymorphic sites per individual in non-broad and broad neutralizers at one month post-diagnosis. The same is shown for sequences sampled at (E-H) six months post-diagnosis and (I-L) three years post-diagnosis. Infections with a single founder are indicated in grey and with multiple founders in pink. P-values for pairwise comparisons (Mann-Whitney U test) between non-broad and broad neutralizers and between broad neutralizers with a single founder infection or multiple founder infections are indicated above pairs. (TIF) [file ppat.1010369.s010.tif]

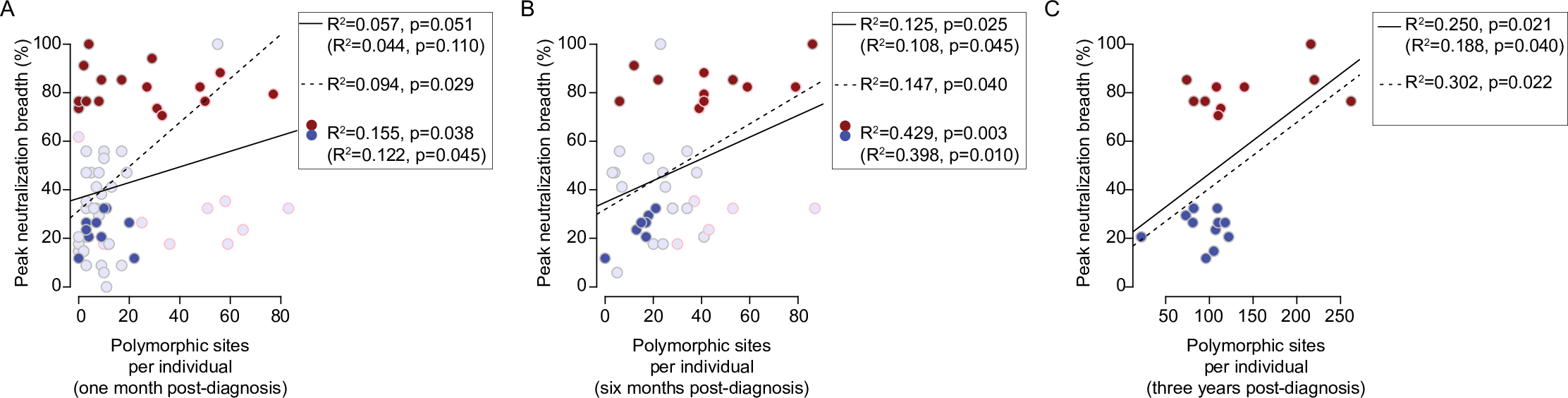

Supplement: S7 Fig — Non-broad neutralizers (indicated in blue) are defined as individuals who neutralized <35% of a 34-virus panel three to four years post-diagnosis and broad neutralizers (indicated in red) as individuals who neutralized >70% of the panel. Individuals sampled less than two years post-infection (intermediate neutralizers) are also shown (indicated in lavender). Participants with a single founder infection are indicated with a grey border and with multiple founder infections with a pink border. Peak neutralization breadth as a function of the number of polymorphic sites per individual at (A) one month, (B) six months, and (C) three years post-diagnosis. Best-fit regression lines are shown for all participants (solid) and for only participants with a single founder infection (dashed). Legends provide best-fit regression statistics for all participants (solid line), only participants with a single founder infection (dashed), and only participants that were non-broad or broad neutralizers (red, blue circles); parenthetical statistics are shown for analyses without individuals with superinfections. (TIF) [file ppat.1010369.s011.tif]
